# Acquired cross-linker resistance associated with a novel spliced BRCA2 protein variant for molecular phenotyping of BRCA2 disruption

**DOI:** 10.1038/cddis.2017.264

**Published:** 2017-06-15

**Authors:** Stefan Meyer, Adam Stevens, Roberto Paredes, Marion Schneider, Michael J Walker, Andrew J K Williamson, Maria-Belen Gonzalez-Sanchez, Stephanie Smetsers, Vineet Dalal, Hsiang Ying Teng, Daniel J White, Sam Taylor, Joanne Muter, Andrew Pierce, Chiara de Leonibus, Davy A P Rockx, Martin A Rooimans, Elaine Spooncer, Stacey Stauffer, Kajal Biswas, Barbara Godthelp, Josephine Dorsman, Peter E Clayton, Shyam K Sharan, Anthony D Whetton

**Affiliations:** 1 Stem Cell & Leukaemia Proteomics Laboratory, Manchester Cancer Research Centre, Division of Molecular and Clinical Cancer Sciences, Faculty of Biology, Medicine & Health, University of Manchester, Manchester, UK; 2 Manchester Academic Health Science Centre, Manchester, UK; 3 Department of Paediatric and Adolescent Oncology, Royal Manchester Children’s Hospital, Manchester, UK; 4 Young Oncology Unit, Christie Hospital, Manchester, UK; 5 Department of Paediatric Endocrinology, Faculty of Biology, Medicine & Health, University of Manchester, Manchester, UK; 6 Department of Clinical Genetics, Section Oncogenetics, VU University Medical Center, Amsterdam, The Netherlands; 7 Mouse Cancer Genetics Program; Center for Cancer Research; Frederick National Laboratory for Cancer Research; National Cancer Institute, Frederick, MD, USA; 8 Department of Toxicogenetics, Leiden University Medical Center, Leiden, The Netherlands; 9 Stoller Biomarker Discovery Centre, University of Manchester, Manchester, UK

## Abstract

*BRCA2* encodes a protein with a fundamental role in homologous recombination that is essential for normal development. Carrier status of mutations *in BRCA2* is associated with familial breast and ovarian cancer, while bi-allelic *BRCA2* mutations can cause Fanconi anemia (FA), a cancer predisposition syndrome with cellular cross-linker hypersensitivity. Cancers associated with *BRCA2* mutations can acquire chemo-resistance on relapse. We modeled acquired cross-linker resistance with an FA-derived *BRCA2-*mutated acute myeloid leukemia (AML) platform. Associated with acquired cross-linker resistance was the expression of a functional BRCA2 protein variant lacking exon 5 and exon 7 (*BRCA2*^*ΔE5+7*^), implying a role for *BRCA2* splicing for acquired chemo-resistance. Integrated network analysis of transcriptomic and proteomic differences for phenotyping of BRCA2 disruption infers impact on transcription and chromatin remodeling in addition to the DNA damage response. The striking overlap with transcriptional profiles of FA patient hematopoiesis and *BRCA* mutation associated ovarian cancer helps define and explicate the ‘*BRCAness’* profile.

BRCA2 encodes a protein with a critical role in homologous recombination and the DNA damage response that is essential for normal development and cancer prevention. *BRCA2* mutation carrier status is linked to familial breast and ovarian cancer (HBOC).^1^ Bi-allelic mutations in *BRCA2* can cause Fanconi anemia (FA), an inherited disease with cancer predisposition and cellular cross-linker sensitivity.^2, 3, 4, 5^ The broad spectrum of cancers associated with FA caused by *BRCA2* mutations infers a critical role of BRCA2 for normal development.^4^ Cancers in *BRCA2* mutation carriers have characteristic features, including distinct gene expression patterns that predict chemo-responsiveness,^6^ and sensitivity to PARP-inhibition, applying the concept of synthetic lethality for targeted therapy.^7, 8^ However, chemo-sensitivity can be lost at relapse.^9^ From one of the few individuals with BRCA2 disruption-associated FA with mutations IVS7+2T>G (*c.864* +*2T>G)* and 3827delGT an acute myeloid leukemia (AML) cell line was established.^10^ While the IVS7+2T>G mutation causes skipping of exon 7 and generates a premature stop codon,^11^ it appears this mutation confers viability in humans due to the expression of the splice variant *BRCA2*^*ΔE4-7*^, which lacks exons 4–7, but retains important BRCA2 functional properties.^12, 13^ In FA patients, *BRCA2*^*ΔE4-7*^ expression associated with the IVS7+2T>G mutation is therefore compatible with fetal viability, but does not prevent severe clinical manifestations of FA, characteristic cellular mitomycin C (MMC) hypersensitivity or malignant transformation, as nearly all patients with IVS7+2T>G develop acute myeloid leukemia (AML) early in life.^4^ In addition, the cultured FA-derived AML cells with IVS7+2T>G with low expression levels of the *BRCA2*^*ΔE4-7*^ transcript maintain MMC hypersensitivity.^10, 12^ Here we use this platform for modeling clonally acquired chemo-resistance. Phenotypic reversion and acquired MMC resistance in these cells is associated with a novel spliced BRCA2 transcript in the MMC-resistant derivate clone, which generates a novel and functional BRCA2 protein variant. This platform is used for further delineation of the ‘*BRCAness*’ phenotype using transcriptomic, proteomic and phosphoproteomic differences in comparison to acquired cross-linker resistance. Validation of our model with data from clinical samples, using expression profiles of bone marrow from FA patients and the ‘*BRCAness’* signature of ovarian cancer, carries important clinical and biological implications.

## Results

### Generation of an MMC-resistant BRCA2-FA-derived AML cell line

**Figure 1:**
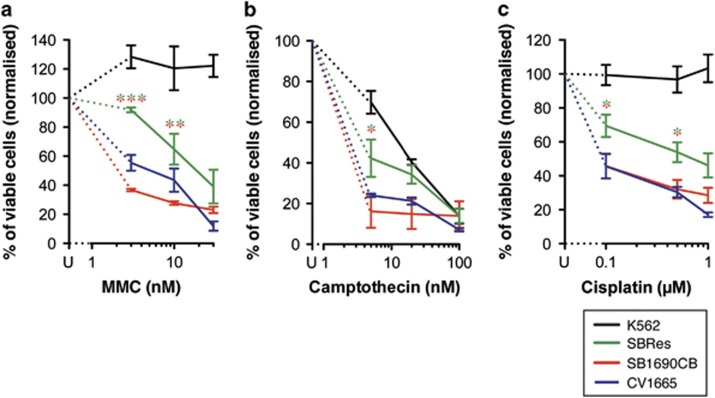
(**a**) MMC growth inhibition. FA-derived AML cells SB1690CB and progeny cell line SBRes were grown with increasing MMC concentrations and without MMC, and counted when untreated cultures had undergone three population doublings. Cell cultures with an IC50 of 10 nM or less were considered MMC sensitive in the FA range. Dose response analysis of sensitive and resistant cells to camptothecin (**b**) and cisplatin (**c**). Statistical analysis: Two way-ANOVA and Bonferroni post-test, *P<0.05. **P<0.01, ***P<0.001. *FANCD2*-mutated FA-disrupted CB1665 cells, and BRCA2-competent K562 cells were used as controls

The MMC-sensitive FA patient-derived AML cell line SB1690CB (*BRCA2* mutations IVS7+2T>G and 3827delGT^10^) was grown in soft gel colony forming assays in the presence of MMC (10–30 nM). In keeping with the FA-phenotype, soft gel colonies only formed under low oxygen tension.^14, 15^ A clonal population of MMC-resistant cells was derived in the presence of 10 nM MMC, but not with higher MMC concentrations. The derived cell line was significantly more MMC-resistant than the parental SB1690CB cells, which are MMC sensitive in the range anticipated for FA cells, similar to FA-control cells CV1665 (Figure 1a). The clonally derived MMC-resistant derivate line showed MMC sensitivity with IC_50_ above the FA range, but displayed higher MMC sensitivity than BRCA2 competent K562 control cells. This new derivate resistant cell line was termed SBRes. To investigate if the acquired MMC resistance also correlates with resistance to other cytotoxic agents, we determined also sensitivity to camptothecin and cisplatin, and showed significantly reduced sensitivity to both compounds in SBRes compared with SB1690CB cells (Figures 1b and c).

### Acquired MMC resistance is associated with increased levels of RAD51 foci formation

**Figure 2:**
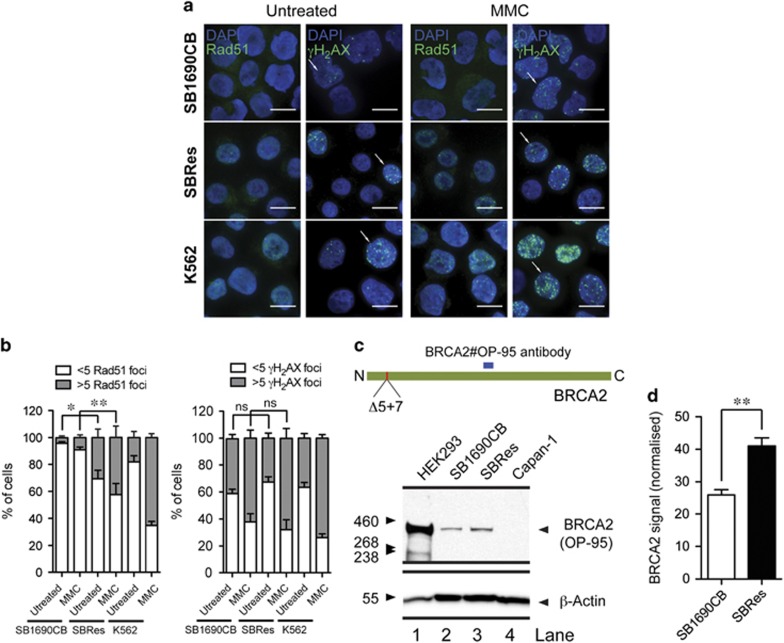
(**a**) Immunofluorescence (IF) analysis of RAD51. RAD1 foci induction by IF after MMC treatment in MMC sensitive SB1690CB, MMC-resistant SBRes and BRCA2 competent K562 control cells. Absence of foci in SB1690CB, and presence of foci in SBRes and control K562 cells as indicated. *γ*H2AX foci formation as control for DNA damage. (**b**) Numerical evaluation of RAD51 foci induction after MMC comparing sensitive SB1690CB cells and resistant SBRes cells (statistical analysis: One way-ANOVA and Tukey post-test *P<0.05, **P<0.01). Increase of *γ*H2AX foci formation after MMC as control for DNA damage. (**c**) Western blot analysis of BRCA2. Antibody recognizing the BRCA2 epitope as indicated (upper panel) for western blot analysis of BRCA2 in MMC sensitive SB1690CB cells, SRes cells with acquired MMC résistance. HEK293 as BRCA2-WT and CAPAN1 cells as negative control for full-length BRCA2. (**d**) Quantification of BRCA2 signal from four biological repeats (statistical analysis: *T*-test, **P<0.01)

To investigate mechanisms of acquired MMC resistance in the SBRes cells, we examined RAD51 foci formation.^16^ Significantly more RAD51 foci were formed in the resistant SBRes cells compared with parental SB1690CB cells, both spontaneously and also after MMC induced damage (Figure 2a). Although the increase in the number of RAD51 foci after MMC treatment was not as high as in K562 control cells, the significantly increased number of RAD51 foci in SBRes cells infers activity of a partially functional BRCA2 protein (Figure 2b).^16^ Using the standard antibody BRCA2 antibody against an internal (mid primary sequence) BRCA2 domain (Figure 2c),^9^ on high exposure in SB1690CB cells and SBRes cells a BRCA2 signal was detected of the same size, but not intensity, as in HEK293 controls (Figure 2c). However, the signal in the SBRes cells was significantly stronger than the signal in the MMC-sensitive SB1690CB cells (Figure 2d), which we presume results from the residual *BRCA2*^*ΔE4-7*^ transcript in these cells.^12^

### Acquired MMC resistance is associated with expression of the novel BRCA2^ΔE5+7^ splice variant

**Figure 3:**
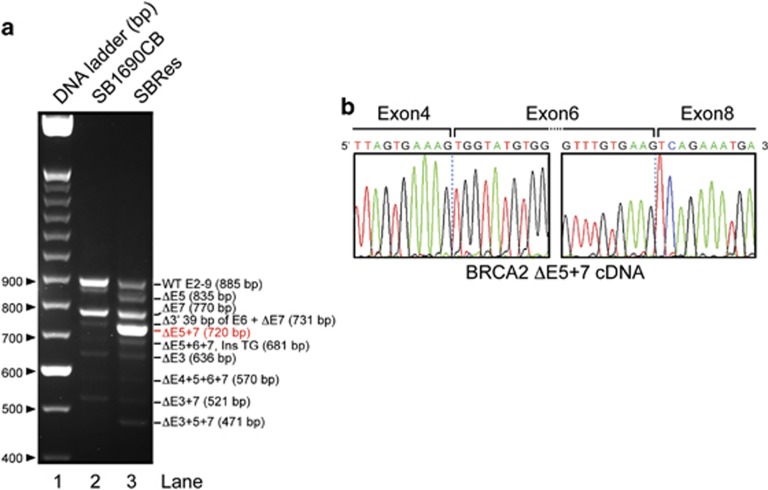
(**a**) Electrophoresis of cDNA amplicons of IVS7 transcripts. Primer oligonucleotides flanking exon 2 and 9 were used to amplify cDNA from SB1690CB (right lane)^12^ and MMC-resistant pedigree SBRes (middle lane). Additional band (Del E5+7), indicating an additional dominant transcript in SBRes, and a dominant smaller transcript in SB1690CB, Del 4–7. (**b**) cDNA sequence of BRCA2^Δ5+7^ showing exon/exon boundaries between exon 4 and 6, and 6 and 8

To investigate the genetic changes associated with apparent BRCA2 competence, we confirmed the presence of IVS7+2T>G and 3827delGT mutations in both cell lines, but found no additional sequence change in genomic DNA that could explain the gained BRCA2 function evident in the resistant SBRes cells (data not shown). As alternative splicing associated with the IVS7+2T>G mutation has been reported to contribute to fetal viability in compound heterozygous FA-D1 patients,^12, 13^ we sequenced *BRCA2* transcripts associated with the IVS7+2T>G mutation. Rather than increased expression of the BRCA2^Δ4-7^, which has been shown to confer some BRCA2 function,^12^ in the resistant SBRes cells two additional strong bands were detected on electrophoretic separation of the RT-PCR amplicons generated with primers targeting cDNA at exon 2 and 9 (Figure 3a). In both cell lines also other previously reported transcripts were detected.^12^ The dominant transcript in SBRes cells, which was not detected in sensitive SB1690CB cells, has a deletion of exons 5 and 7, *BRCA2*^*ΔE5+7*^ (Figure 3b). This transcript has an in-frame deletion of 165 base pairs compared with WT-BRCA2. Using BioEdit Protein prediction software, the BRCA2 protein translated from the *BRCA2*^*ΔE5+7*^ transcript would have 3363 amino acids (aa) with an internal deletion of 55 aa, an altered aa sequence translated from exon six and otherwise conserved functional domains, consistent with the signal size on western blotting (Figure 2c). The other strong band with electrophoretic separation of BRCA2 transcripts (Figure 3a), detected only in the SBRes cells, corresponds to a transcript lacking exon 5 (*BRCA2*^*ΔE5*^). This transcript was previously described, associated with the c.475+1G>A variant, and classified as deleterious.^17^

### BRCA2^ΔE5+7^ corrects BRCA2 disruption in mouse ES cells

**Figure 4:**
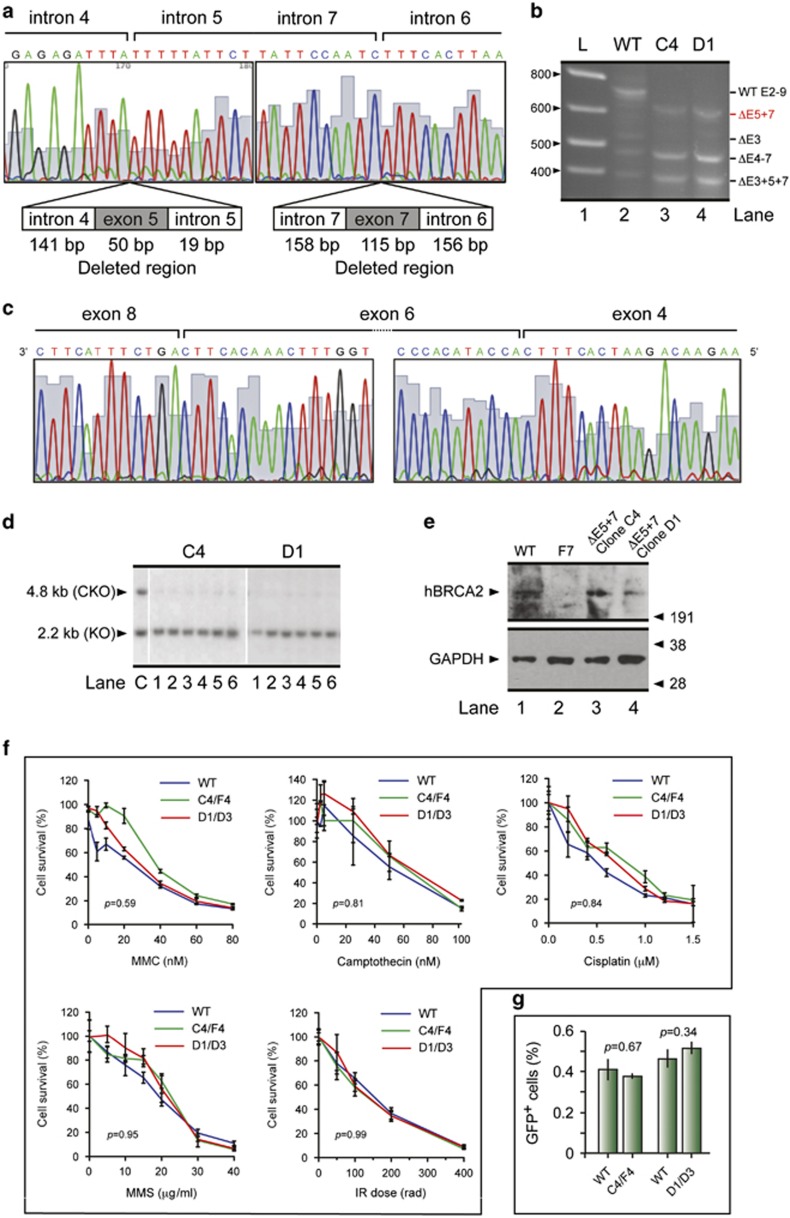
BRCA2 ^Δ5+7^ expression and functional analysis in mouse ES cells. (**a**) Sequence of the region containing exons 5 and 7 and the flanking intronic regions deleted from *BRCA2*. (**b**) RT-PCR analysis of *BRCA2* transcripts in mouse ES cells expressing WT and Δ5+7 BRCA2 transgene using primers from exons 2 and 9. (**c**) Sequence analysis of the 623bp RT-PCR fragment that lacks exons 5 and 7. (**d**) Southern blot analysis of HAT^r^ ES cell colonies obtained after Cre-mediated deletion of the conditional of BRCA2. The upper band corresponds to the conditional allele (cko), and the lower band corresponds to the mutant allele (ko). The sizes of the bands are shown on the left. (**e**) Detection of conditionally expressed BRCA2 by immunoblot. (**f**) XTT assay of cells expressing WT or Δ5+7 mutant BRCA2 (C4/F4 and D1/D3) following 48 h of treatment with MMC, cisplatin, camptothecin, MMS and *γ*-irradiation (IR) to examine their sensitivity to these DNA-damaging agents. (**g**) Efficiency of homologous recombination (HR) measure by using DR-GFP reporter after generation of I-*Sce*I induced double strand break. Graph shows the percentage of GFP positive cells in WT and two Δ5+7 mutant clones (C4/F4 and D1/D3)

To test the effect of deletion of exons 5 and 7 on BRCA2 function we utilized the mouse embryonic stem (ES) cell-based *in vitro* functional assay developed for comprehensive functional assessment of *BRCA2* variants.^18^ To functionally assess *BRCA2*^*ΔE5+7*^ we deleted exons 5 and 7 in a BAC clone containing full-length human *BRCA2* (Figure 4a) and expressed this in PL2F7 cells engineered to have a mutant and a conditional allele of mouse *Brca2*.^18^ We confirmed the presence of the 5′ and the 3′ end of the *BRCA2* transgene in the ES cells by PCR and also confirmed the expression of the transgene by RT-PCR using primers to exons 11 and 18. We next examined the effect of the deletion of exons 5 and 7 on the expression of various *BRCA2* transcripts by RT-PCR using primers from exon 2 and exon 9 as described previously.^12^ A 788 bp band corresponding to exons 2–9 was observed in the ES cells carrying the wild-type *BRCA2* (WT), which was absent in the two clones (C4 and D1) expressing the mutant *BRCA2* (Figure 4b). Instead, a 623 bp band was observed that was confirmed by sequencing to represent a transcript lacking exons 5 and 7. In addition, a transcript lacking exon 3 was detected in cells expressing WT-*BRCA2*. Two additional bands, 474 bp and 374 bp in size, corresponding to transcripts lacking exons 4–7 and lacking exons 3, 5 and 7, respectively were detected in all samples. All fragments were sequenced to confirm their identity (Figure 4c). We next examined the effect of lack of exons 5 and 7 (BRCA2^*Δ5+7*^) on key biological functions of BRCA2. We first tested its ability to rescue the lethality of *Brca2*-null mouse ES cells. We deleted the conditional allele of *BRCA2* and selected the recombinant expressors in HAT media as described previously.^18^ We obtained HAT resistant clones that were confirmed by Southern blot analysis to be *Brca2-null* (Figure 4d). The number of HAT colonies on plates expressing mutant *BRCA2*^*Δ5+7*^ was comparable to the cells expressing WT-BRCA2 (data not shown). We confirmed the expression of BRCA2^Δ5+7^ in ES cell clones and found it to be at levels comparable to WT-BRCA2 (Figure 4e). We next tested the DNA repair function of BRCA2^Δ5+7^. ES cells expressing WT and *BRCA2*^*Δ5+7*^ were treated with MMC, camptothecin, cisplatin, methylmethane sulfonate (MMS) and irradiation (IR), and cell survival was measured after 48 h (Figure 4f). We found no statistically significant difference between WT and the two mutant clones in their response to these cytotoxics. Next, we examined the effect of loss of exon 5 and 7 on homologous recombination (HR), a key function of BRCA2. We used the DR-GFP-based reporter integrated into the *Pim1* locus in the PLF7 ES cells.^3^ We measured HR efficiency by generating an I-*Sce*I restriction enzyme-induced double strand break (DSB) into a non-functional GFP reporter gene. A functional GFP is generated if the DSB is repaired using a promoterless but functional GFP gene placed upstream of the restriction site. The percentage of GFP positive cells obtained from both mutant clones was similar to WT expressing ES cells (Figure 4g), suggesting that the mutant BRCA2^Δ5+7^ is fully proficient in HR. Taken together; these results show that the BRCA2^Δ5+7^ protein encoded by transcripts lacking exons 5 and 7 is functionally indistinguishable from WT.

### Detection of the BRCA2^Δ5+7^-encoded protein

**Figure 5:**
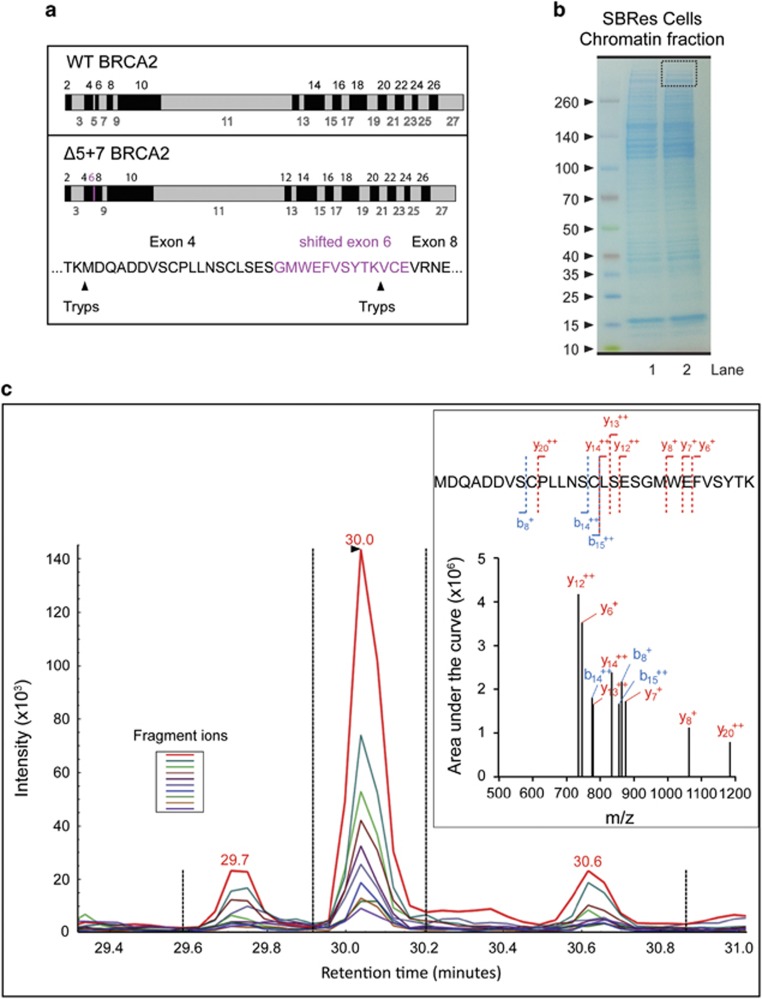
Mass spectrometric detection of the BRCA2^Δ5+7^ protein. (**a**) Schematic illustration of WT-BRCA2 (upper panel) and BRCA2^Δ5+7^ protein (lower panel) with putative unique trypsin digestion sites. Amino acid sequence derived for the shifted reading frame of the exon 6 is indicated in purple. (**b**) Gel electrophoresis and gel slice selection (dotted square) of chromatin fractions of SBRes used for trypsin digestion, tryptic peptides isolation and subsequent mass spectrometry analysis. (**c**) Targeted mass spectrometry SRM spectrum for the splice variant-specific peptide MDQADDVSCPLLNSCLSESGMWEFVSYTK detecting ten co-eluting fragment ions (color coded lines, left insert). A reconstituted spectrum using the SRM data is also shown (right insert). b ions (blue) and y ions (red) are annotated related to the peptide sequence

To investigate the presence of a BRCA2 protein that lacks the amino acid encoded by exons 5 and 7 in the resistant cells, we carried out targeted mass spectrometric analysis of chromatin fractions of SBRes cells from gel slices containing proteins >350kDa. We employed selected reaction monitoring mass spectrometry for specific tryptic hydrolytic peptides of the truncated BRCA2 protein variant,^19^ taking advantage of a novel putative trypsin digestion site (aa sequence TKV, Figure 5a) in the *BRCA2*^*ΔE5+7*^-encoded protein (Figure 5b). A tryptic hydrolytic peptide with the specific aa sequence for the BRCA2^*ΔE5+7*^ protein was detected in tryptic digests from gel fractions >350kDa. The identified peptide also covered the exon/exon transition between exon 6 and 8, further confirming the presence of a high molecular weight truncated BRCA2 protein variant containing aa sequence encoded by the shifted exon 6, but lacking exon 7 (Figure 5c), which is not present in other proteins, thus proving the expression of the BRCA2^*ΔE5+7*^ protein variant.

### Molecular phenotype delineation of BRCA2 disruption and acquired chemo-resistance

**Figure 6:**
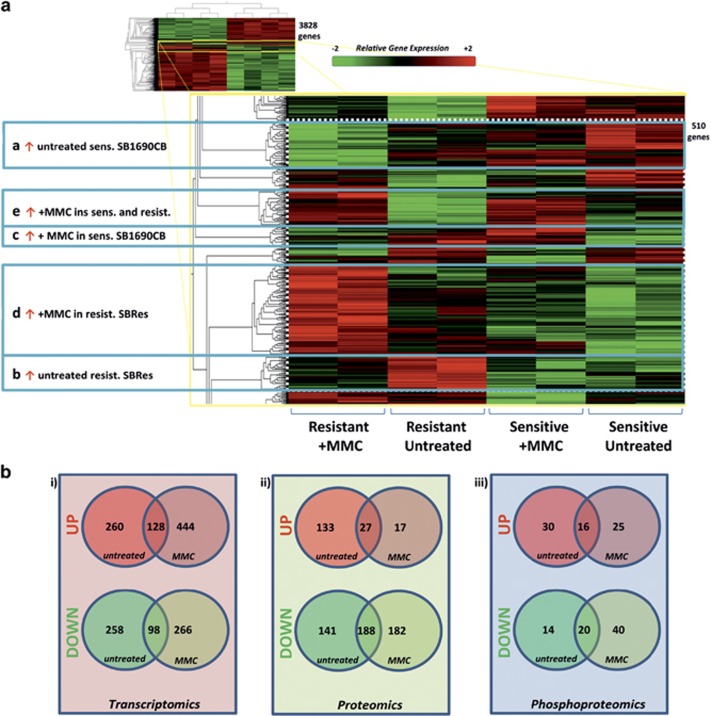
Expression phenotype of acquired MMC resistance. (**a**) Heat map illustration of differential gene expression between MMC-resistant and MMC-sensitive cells with and without MMC treatment (2MMol). 3828 differentially expressed genes (group ANOVA *P*<0.05) were identified. Clustering of the data within the heat map delineated distinct groups of gene expression patterns differentiating the MMC-resistant and sensitive states as indicated (total of 510 genes). Corresponding lists are provided in Supplementary Tables. (**b**) Numerical Venn diagram illustration of transcriptomic analysis (left, *P*<0.01), proteomic (middle) and phosphoproteomic (right) analysis comparing sensitive and resistant cell lines with and without MMC split into up and downregulated transcripts/proteins/phosphopeptides. Corresponding lists are provided in Supplementary Material

**Figure 7:**
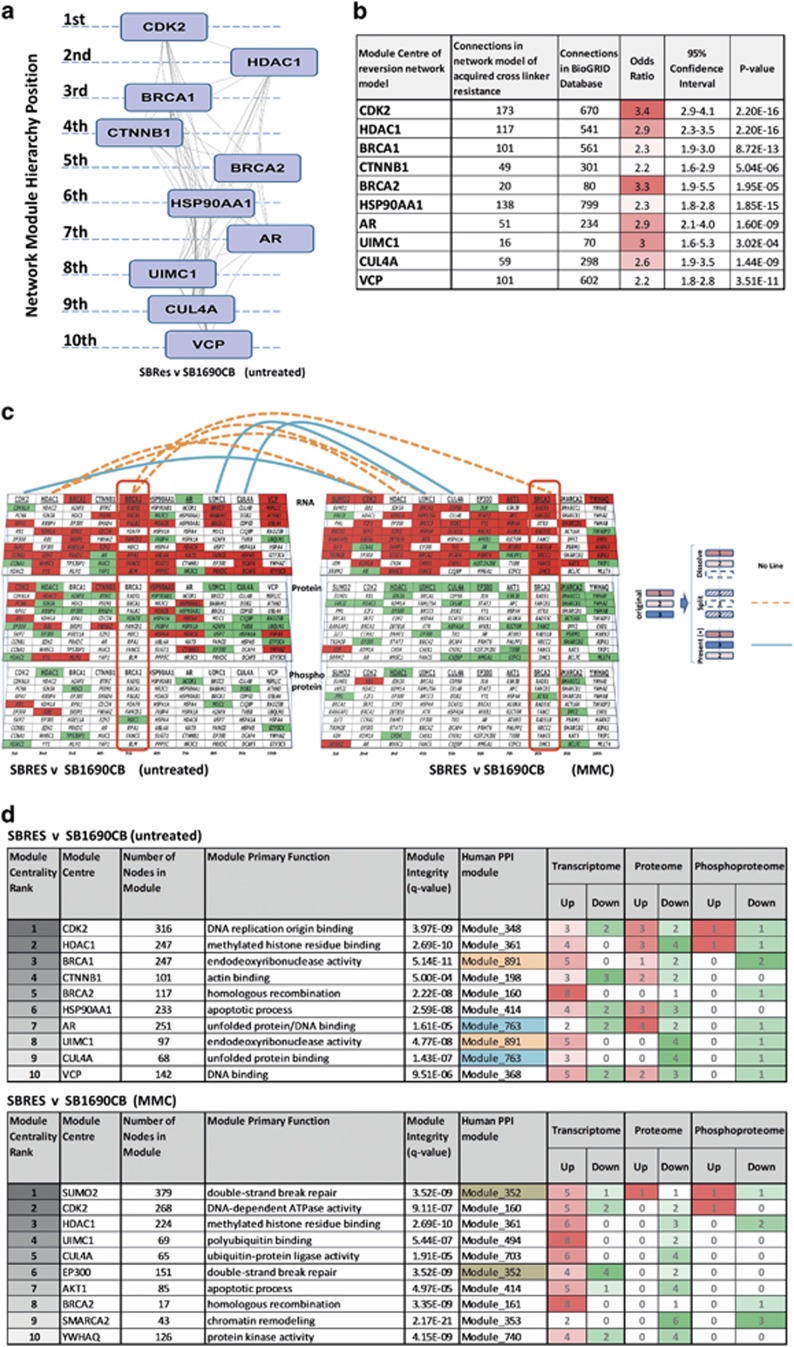
Integrated analysis defining differences between resistant and sensitive cells untreated and in response to Mitomycin C (MMC). (**a**) Network modules delineated by inferred interactions from integrated analysis of transcriptional, proteomic and phosphoproteomic differences between untreated sensitive and resistant cells. The network modules were delineated and ranked by network centrality applying the Moduland community clustering approach. (**b**) Quantitative characterization of network modules delineated from integrated analysis of acquired cross-linker resistance, by defining differences in observed connectivity in relation to the expected connectivity in the human interactome (BioGRID). Odds ratio, 95% confidence intervals and *p*-values generated using Fisher’s exact test. (**c**) Network dimensions and changes in response to MMC. Changes affecting the three dimensions of the integrated data (transcriptome, upper level; proteome, middle level; phosphoproteome, lower level) of untreated cells (left panel) were mapped to the central units of each network module in hierarchical order; downregulated transcription and decreased protein expression/phosphorylation in green; up-regulated transcription, higher levels of protein expression and presence of phosphorylation in red. Cartoon besides panels: In response to environmental changes modules can remain, but the position in the hierarchy can change, split into new clusters or dissolve. The effects on the network of MMC treatment are illustrated (right panel), with delineation of novel clusters dominated by SUMO, AKT1, EP300, YWHAC and SMARCA2, splitting and resoling clusters as indicated. (**d**) Statistical and ontological characterization of clusters and dominating network modules for untreated cells (upper panel) and in response to MMC (lower panel)

Having identified splicing causatively associated with acquired resistance in a BRCA2 mutation associated malignancy, we addressed consequences of acquired cross-linker resistance to validate this AML system for broader clinical application by gene expression analysis. We delineated distinct clusters of differentially expressed transcripts (*P*<0.05) in sensitive and resistant cells, and also in response to low dose (2 mM) MMC (Figure 6a; Supplementary Table 1). To investigate phenotypic network connections associated with transcriptional changes, we focused on changing transcripts of higher significance (*P*<0.01), and complemented this analysis with isobaric tagging relative quantification (ITRAQ) mass spectrometry data, determining (phospho-) proteomic differences between untreated sensitive and resistant cells, untreated and in response to MMC (Figure 6b; Supplementary Table 2A–C). Higher levels of nuclear (RAD51, Catenin beta-1 (*β*-catenin) and Lamin A/C in resistant SBRes cells detected by mass spectrometry were confirmed by western blot (Supplementary Figure 1A). Increased levels of Nibrin phosphorylation at serine 343 after MMC treatment detected by mass spectrometry only in resistant SBRes were repeatedly confirmed by western blot analysis (Supplementary Figure 1B). Integrated computational analysis of transcriptomic, proteomic and phosphoproteomic differences employing a community clustering approach (Moduland)^20^ determined the dominant elements of the interactome. A robust hierarchy of the first ten modules with respect to protein:protein (PPI) interactions was constructed. The centrality of BRCA2 being one of the 10 highest ranking modules provides further evidence that acquired BRCA2 competency is underlying the acquired cross-linker resistance (Figure 7a). Odds ratios for all dominating modules were highly significant, based on comparison of connectivity data of the human interactome (Figure 7b). Genes and proteins of distinct clusters affected by transcriptional, proteomic and phosphoproteomic levels are represented as illustrated for untreated cells (Figure 6c, left panel), and quantitatively assessed (Figure 7d, upper panel). Networks such as illustrated here can be affected by environmental changes. Consequently, clusters can persist, split or dissolve, and new clusters can form (Figure 7c, cartoon illustration beside panels). In response to low dose MMC treatment the BRCA1-dominated cluster in untreated cells dissolved, and the BRCA2-dominated cluster split with 3 components joining the UIMC1 cluster, which ranked higher in the MMC response. Five *de novo* network modules affected by acquired chemo-resistance formed (Figure 7c, right panel). Novel clusters forming and delineating differences between sensitive and resistant cells in response to MMC treatment were dominated by SUMO2, EP300, AKT1, SMARCA2 and YWHAQ as dominant regulatory network modules (Figure 7c, right panel; 3d lower panel).

### Transcriptional profiles associated with the BRCA2 defect overlap with transcriptional patterns of FA-disrupted hematopoiesis and the BRCAness signature of ovarian cancer

**Figure 8:**
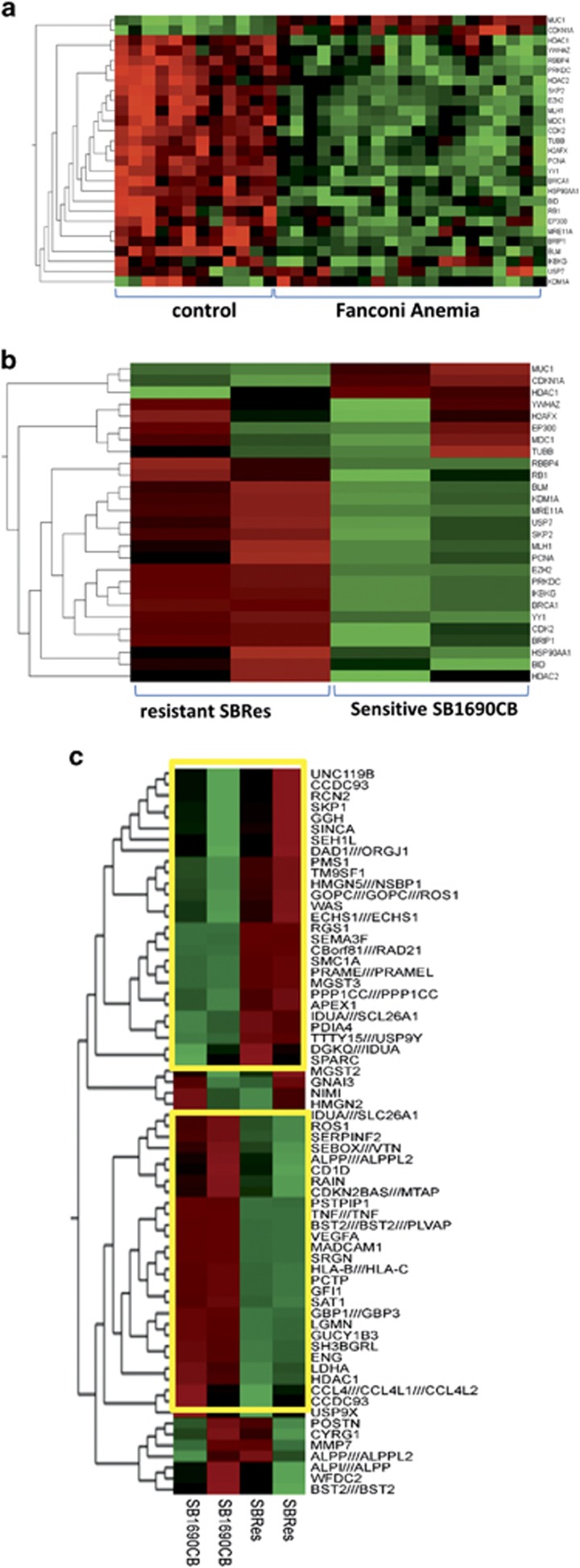
Evaluation of BRCA2 disruption-associated transcriptional phenotype with transcriptional patterns of clinical samples. (**a**) Heat map showing expression of transcripts relating to dominating transcriptional network modules delineated in gene expression data of bone marrow samples from FA patients compared with unaffected controls as GEO data set GSE16334.^21^ (**b**) Analysis of these transcripts in FA-derived MMC sensitive SB1690CB cells and their resistant SBRes progeny. (**c**) Heat map showing BRCA disruption-associated genes in clinical ovarian cancer^6^ in sensitive SB1690CB Cells compared with their resistant SBRes progeny. Significantly differentially expressed transcripts framed in yellow boxes

Having molecularly phenotyped an *in vitro* model of acquired cross-linker resistance, we validated our findings with clinical data. As cross-linker hypersensitivity is also a feature of cells with mutations in other FA-genes, we explored to what extent our FA-derived AML model is corresponding to transcriptional profiles in FA-disrupted compared with normal hematopoiesis. We analyzed transcriptional profiles of bone marrow samples from 22 FA patients compared with unaffected controls,^21^ again applying the Moduland algorithm in Cytoscape.^20^ The analysis of the clinical FA bone marrow samples identified 153 network modules dominating the transcriptional differences (Supplementary Figure 2). Comparing these with the networks determined in our FA-derived AML model, 27 common network modules were identified (*P*<0.001, hypergeometric test). We then compared the expression of genes related to these 27 dominating network modules in FA compared with normal hematopoiesis. Difference in expression levels of these genes was highly correlated with the FA-defect (Figure 8a). We then determined the expression of these 27 associated genes in the AML model, in which we independently also identified the HDAC1, BRCA1 and CDK2 modules affected by BRCA2 disruption. We showed corresponding expression patterns in 22 of the 27 genes (Figure 8b), indicating that gene expression networks in clinical FA bone marrow samples and our AML model are strongly and similarly influenced by the FA-associated cross-linker repair defect. To validate our model further, we investigated the gene expression signature of *BRCA* mutation associated ovarian cancer that predicts response to chemotherapy^6^ in our FA-derived *BRCA2* mutation associated AML model. Of the 60 differentially expressed genes that delineate BRCA disruption and chemo-responsiveness in ovarian cancer, 54 are also highly significantly differentially expressed in SBRes compared with the MMC sensitive SB1690CB (Figure 8c), inferring that parts of the transcriptional ‘BRCA*ness* signature’ may be tissue independent.

## Discussion

The cancer spectrum associated with *BRCA2* mutations in HBOC and FA-D1 infers a fundamental role of BRCA2 for normal development and prevention of malignant transformation by its essential functions in homologous recombination and the DNA damage response.^22^ The cellular phenotype associated with BRCA2 disruption includes the FA-characteristic cross-linker sensitivity, the basis for targeted therapeutic approaches applying the concept of synthetic lethality.^8^ Further characterization of causes and consequences of BRCA2 disruption can therefore aid the identification of ‘*BRCAness*’ or other defects in the DNA damage response in sporadic tumors relevant for targeted therapy, and might further clarify functional aspects of BRCA2. With AML cells derived from one of the few reported individuals with bi-allelic *BRCA2* mutations,^10^ we modeled acquired cross-linker resistance in order to address these clinically important issues. Associated with acquired cross- linker resistance we identified a novel *BRCA2* splice variant, which retains functional aspects of *BRCA2* relevant for DNA repair. The expression of the *BRCA2*^*ΔE5+7*^ transcript, possibly caused by modulation of a splicing factor, deep intronic or epigenetic changes, but not from a change in the coding region of the mutated *BRCA2* gene itself, evaded detection via *BRCA2* sequencing of exons and exon-intron boundaries. This is adding to a growing body of data inferring clinical relevance of *BRCA2* splicing. While many BRCA2 splice variants have been linked to distinct *BRCA2* mutations,^12, 13, 17, 23, 24, 25, 26^ our findings of the splice variant *BRCA2*^*ΔE5*^, which previously has only been described associated with the c.475+1G>A variant,^17^ and the novel *BRCA2*^*ΔE5+7*^ in the revertant SBRes cells infers a more general role of *BRCA2* splicing in normal and malignant tissue, of which regulation is only partially understood. However, an integral role of splicing in double strand repair and chemo-resistance is increasingly recognized.^27, 28, 29^ Detection of unique peptide sequences of the altered BRCA2 protein by mass spectrometry provides not only first evidence of translation of a *BRCA2* splice variant, but also some novel insights into BRCA2 protein function. The 55 aa deletion of the BRCA2^*ΔE5+7*^ protein includes several PLK phosphorylation sites, which have been implicated in cellular localization of BRCA2.^30^ Our results infer that these phosphorylation events are not essential for abrogation of MMC-sensitivity *in vitro*, but further investigations into regulation of localization, stability and mobility of the BRCA2^*ΔE5+7*^ protein would be important to understand details of the functional relevance of the affected BRCA2 protein domains. From a clinical perspective it will be important to investigate if alternative splicing may be involved in a subset of chemo-resistant *BRCA2* mutation associated tumors for which the underlying mechanism cannot be identified by analysis of the *BRCA2* coding sequence of the genomic DNA.^9, 31, 32, 33^ Having identified a novel *BRCA2* splice isoform associated with phenotypic reversion, we confirmed previously reported data with respect to single observations in *BRCA2* mutation associated tumors, such as dependence of BRCA2 function for nuclear accumulation of RAD51,^34, 35^ or impaired phosphorylation of Nibrin, which has been previously observed associated with defects in the FA/BRCA pathway,^36^ by mass spectrometry and western blot analysis. The acquisition of systems biology data with transcriptomic, proteomic and phosphoproteomic details provides additional candidates that can, alone or in combination, be evaluated as biomarkers for cross-linker sensitivity. Importantly, these data also allow the further characterization of the ‘BRCA*ness’* phenotype by integrative network analysis of large datasets generated from *BRCA2* mutation associated cancer cells with acquired resistance. The delineation of BRCA2 as a module dominating one of the highest ranking clusters in the network determining the difference between sensitive SB1690CB cells and the resistant progeny strongly infers that the underlying mechanism for cross-linker resistance is gain of BRCA2 function, which is supported experimentally by the demonstration of RAD51 foci in the SBRes cells with acquired resistance. Other dominant modules determining differences associated with acquired cross-linker resistance are closely linked to BRCA2 function in the DNA damage response, such as BRCA1, which functions in the FA/BRCA pathway,^37, 38^ or interacting with the FA/BRCA pathway such as CTNNB1^39^ and UIMC1.^40^ An effect of BRCA2 dysfunction on CDK2 is supported by recent experimental evidence of a direct phosphorylation of BRCA2 by CDK2.^41^ CUL4A ubiquitin ligase has been suggested to be a marker for impaired DNA damage response,^42^ and the high ranking of CUL4A implies the probability of a much more involved connection to the FA/BRCA DNA damage response pathway. A similar hypothesis can be developed for the androgen receptor AR, HDAC1, which infers an effect on chromatin remodeling and transcription, and HSP90A1. The delineation of SUMO2 as a novel dominating module in the difference of the MMC response is in line with the recent recognition of a major role of SUMO2 in the DNA damaged response.^43, 44^ Clinical evaluation of our model by comparison with gene expression of FA-disrupted haematopoiesis using an innovative computational approach that allowed comparison of *in vitro* with clinical data, revealed a significant overlap with respect to modules dominating gene expression patterns. Our findings suggest that the FA/BRCA pathway has a common effect on transcriptional patterns in normal as well as malignant haematopoiesis. In particular, expression of specific transcriptional regulators such as YY1 and EZH2 appears to be dependent on the presence of a functional FA/BRCA pathway and further support an effect of the FA/BRCA pathway on transcription. In addition, the BRCA disruption-associated expression signature of epithelial ovarian malignancies is strikingly mirrored in our model. Our finding that of the 60 signature transcripts for ‘*BRCAness*’ in ovarian cancer,^6^ 54 (90%) are also differentially expressed in sensitive SB1690CB cell compared with resistant SBRes cells implies that a ‘*BRCAness*’ signature might be to some extent tissue independent in cancer. Data from this study can form the basis of further clinical evaluation.

Our study supports a role of *BRCA2* splicing for acquired chemo-resistance in *BRCA2* mutation associated malignancy by providing functional data of a variant BRCA2 protein, which we detect with targeted mass spectrometry. Phenotyping by integrated analysis of acquired chemo-resistance infers impact of the BRCA2 defect on chromatin remodeling and transcription in addition to other DNA damage response elements. Evaluation of this model with clinical data shows strong overlap with FA-hematopoiesis and BRCA2 associated ovarian cancer.

## Materials and Methods

### Cell lines and culture

The AML cell line SB1690CB with *BRCA2* mutations IVS7+2T>G and 3827delGT was maintained mycoplasma free as described.^10, 45^ An MMC-resistant derivative was generated by MMC exposure in concentrations ranging from 10 to 30 nM, at normal and low (5%) oxygen. Surviving cells were plated in semi-solid methylcellulose based medium (Methocult; Stem Cell Technologies, Cambridge, UK) with the same MMC concentrations. Colonies grew only under low oxygen conditions and with 10 nM of MMC.^14, 15^ Identity of the origin of both resistant and sensitive cells was confirmed using the Promega PowerPlex 16 System (Promega, Southampton, UK). MMC sensitivity of expanded cultures was determined by MMC growth inhibition as described previously.^10, 46^ The FA-derived lymphoblastoid cell line CV1665 (*FANCD2* mutated^47^), and the *BRCA2* disrupted pancreatic carcinoma cell line CAPAN1 lacking full-length BRCA2 were cultured in 1640RPMI with 10% FCS and used as a MMC sensitive, and the myeloid leukemia cell line K562 as MMC-resistant control. For WB experiments HEK293 cells were used as full-length BRCA2 controls. For MMC treatment cells were exposed to 2 mM MMC for one hour, washed, and processed after 8 h recovery.

### Molecular biology and imaging

Antibodies: For BRCA2 detection we employed the standard mouse monoclonal antibody OP-95 (BRCA2-ab1) raised against an internal domain (aa1651–1821) (EMD Millipore, Livingston, UK).^9^ Anti-mouse HRP conjugated antibody (GE Healthcare, Little Chalfont, UK) was used as secondary antibody. WB was carried out with protein loading of 70 μg/well, resolved in NuPAGE™ 3–8% Tris-Acetate Protein Gels (Invitrogen, Thermofisher, Hemel Hempstead, UK). For quantitation of WB signals from biological replicates (*n*=4) we used the GelDoc XRS System (Bio-Rad, Watford, UK) and Image J software. In brief, raw digitally acquired image files were processed using HiLo lookup table (LUT) to avoid saturated pixels. Regions of interests (ROIs) were created for each band and the integrated signal density was calculated, normalized against the loading control, plotted and analyzed using Prism 5 (GraphPad Software, La Jolla, CA, USA).^48^ Normalized signal intensity values were compared by T-test. For RAD51 immunofluorescence we used the antibody H-92, sc-8349 (Santa Cruz Biotechnology, Heidelberg, Germany) as described previously,^16^ and the phospho-Histone H2AX (Ser139, a.k.a. *γ*H_2_AX) rabbit mAb (Cell Signaling Technology, Leiden, The Netherlands, 20E3, #9718). Anti rabbit-IgG Alexa 488 conjugated antibody (Life Technology) was used as secondary antibody. Slides were mounted with Prolong Gold anti-fade plus DAPI (Cell Signaling Technology). Representative images were acquired using an Olympus BX51 microscope, UPLAN APO 60X objective, coupled with a Retiga 6000 camera (Q-Imagine software, Q-Imagine). For each condition 100 cells were analyzed manually by counting foci. Other antibodies used for Western blot were rabbit anti-pS343-Nibrin (CST), Nibrin (CST), Lamin A/C (CST), CTNNB1 and *β*-Actin (Sigma).Sequencing of *BRCA2* and *BRCA2* cDNA was carried out as reported previously.^10, 12^

### Gene expression analysis

RNA was extracted (QIAgen kit) from two biological replicate of SB1690CB and SBRes cultured under standard conditions and with MMC as above. Concentration and purity of RNA was determined using the NanoDrop spectrophotometer. The WT-Ovation Exon Module V1.0 (NuGEN) was used to generate ST-cDNA and 4 *μ*g was hybridized to Human Exon 1.0 ST (Affymetrix) arrays. Analysis of differential gene expression was performed using the Robust Multichip Average (RMA), pre-adjusted for GC content with quantile normalization followed by a mean probe set and a gene level summarization using Partek Genomics Suite software (version 6.12). The data set generated was subject to quality control to investigate the presence of outliers and further confounding effects using Principal Components Analysis (PCA) and Iso-map multidimensional scaling (MDS) to demonstrate data homogeneity (Qlucore Omics Explorer 2.2). Analysis of variance (ANOVA) was used to determine differential gene expression between groups. Supervised hierarchical clustering was performed on the co-variant normalized data using data normalized to a mean of zero and a variance of one (Partek Genomics Suite software version 6.12). The substitution of randomly selected genes was used to assess the specificity of the clusters observed over ten iterations (QlucoreOmics Explorer). The identification of enriched gene ontology (GO) of biological pathways was performed within WebGestalt using the Identification of biological pathways and functions associated with gene expression changes was undertaken using a right sided Fisher’s exact test within Ingenuity Pathway Analysis software (IPA) and confirmed using the Pathway Commons database applying a hypergeometric test with a Benjamini-Hochberg correction for multiple testing.

### Mass spectrometry

Protein and phosphopeptide quantification was carried out using iTRAQ Mass spectrometric as described previously (Pierce *et al.*, 2008; Unwin *et al.*, 2005; Unwin *et al.*, 2006). BRCA2 peptides were detected using selective reaction monitoring Mass Spectrometry as previously described (Unwin *et al.*^19^). Details can also be found supplementary.

### Functional evaluation of BRCA2 lacking exons 5 and 7 in mouse ES cells

Functional evaluation of *BRCA2*^*ΔE5+7*^ transcript lacking exons 5 and 7 was performed using a mouse ES cell-based assay as described previously.^49^ Briefly, we deleted exons 5 and 7 and a portion of the flanking intronic sequences (141 bp of 3′ end of exon 4, 19 bp of 5′ end of intron 5, 156 bp of 3′ end of intron 6 and 158 bp of 5′ end of intron 7) of human BRCA2 cloned in BAC RP11-777I19 by recombineering using galK-based selection/counter-selection method as described previously.^50, 51^ Mutant BAC DNA was electroporated into PL2F7 mouse ES cells followed by the deletion of the endogenous mouse *Brca2* gene by Cre-mediated recombination.^18^ Recombinant clones lacking endogenous *Brca2* were selected in HAT media as described previously.^18^ For BRCA2 immunodetection total proteins were extracted in IP buffer (20 mM HEPES (pH 7.5), 100 mM NaCl, 1 mM EDTA, 1 mM EGTA, 1 mM NaF, 1 mM DTT, 0.1% Triton X-100, 1 mM PMSF, protease inhibitor cocktail (Roche), phosphatase inhibitor cocktail (Roche)). Human BRCA2 protein was immunoprecipitated by incubating lysates with protein-G agarose beads (Roche) and antibody against c-myc tag (Clontech #631206) overnight at 4 °C. After that beads were washed in lysis buffer for 4-5 times and the immunoprecipitated complex were separated in 4–12% bis-tris gel (Invitrogen) by electrophoresis and BRCA2 protein was detected with c-myc antibody (Cell Signaling #2278). For input control 1/50th of input lysate were separated using 4–12% bis-tris gel and detected with GAPDH antibody (Cell Signaling #2118). We tested the efficiency of the DNA repair function by challenging the recombinant cells with DNA-damaging agents: MMC, camptothecin, methylmethane sulfonate, cisplatin and g-irradiation. Significance of the survival difference between mutant and wild-type cells was assessed by two-tailed t-test. Efficiency of homologous recombination was measured using a GFP-based reporter as described previously.^52, 53^ For the RT-PCR, we synthesized cDNA using RNA isolated from mouse ES cells expressing either a wild-type or mutant human BRCA2 gene using a SuperScript III reverse transcriptase (Invitrogen). PCR was performed using exon 2 (Forward) (5′-GCATTGGAGGAATATCGTAGG-3′) and exon 9 (reverse) (5′-TTCACTGTCTGTCACAGAAGC-3′) primers as described previously.^12^

### Systems biology and integrated data analysis

Global gene expression profiling by exon array together with ITRAQ proteome and phosphoproteomic analysis was subjected to an integrated network analysis using an interactome model. Network analysis allows the identification and prioritization of key functional elements within interactome models. To derive an interactome model, differentially expressed genes in a combined ‘omic’ data set were used as ‘seeds’, and all known protein:protein and protein:genetic interactions between the seeds and their inferred immediate neighbors were calculated to generate a biological network using the BioGRID model of the human Interactome (31.2.103). The robustness of module integrity was confirmed by comparison with databases of human PPI^54^ (module integrity *P*-value; Figure 3g).Network generation and processing was performed using Cytoscape 2.8.3. Clustering and ‘community structure’ of modules within biological networks are known to be associated with function. To prioritize these functional components within interactome models we used the ModuLand plugin for Cytoscape 2.8.3^20^ to determine overlapping modules, and to identify hierarchical structures within the model, thus enabling the identification of key network elements using the hypergeometric test with a Benjamini-Hochberg false discovery rate correction. This analysis was carried out with data generated as part of this study and with the GEO data set GSE16334.^21^
