# Supplementary figures and images for: Acquired cross-linker resistance associated with a novel spliced BRCA2 protein variant for molecular phenotyping of BRCA2 disruption
Source: Cell Death Dis. 2017 Jun 15;8(6):e2875–. doi: 10.1038/cddis.2017.264 (PMC5520920; doi:10.1038/cddis.2017.264)

Suppl. Figure 1

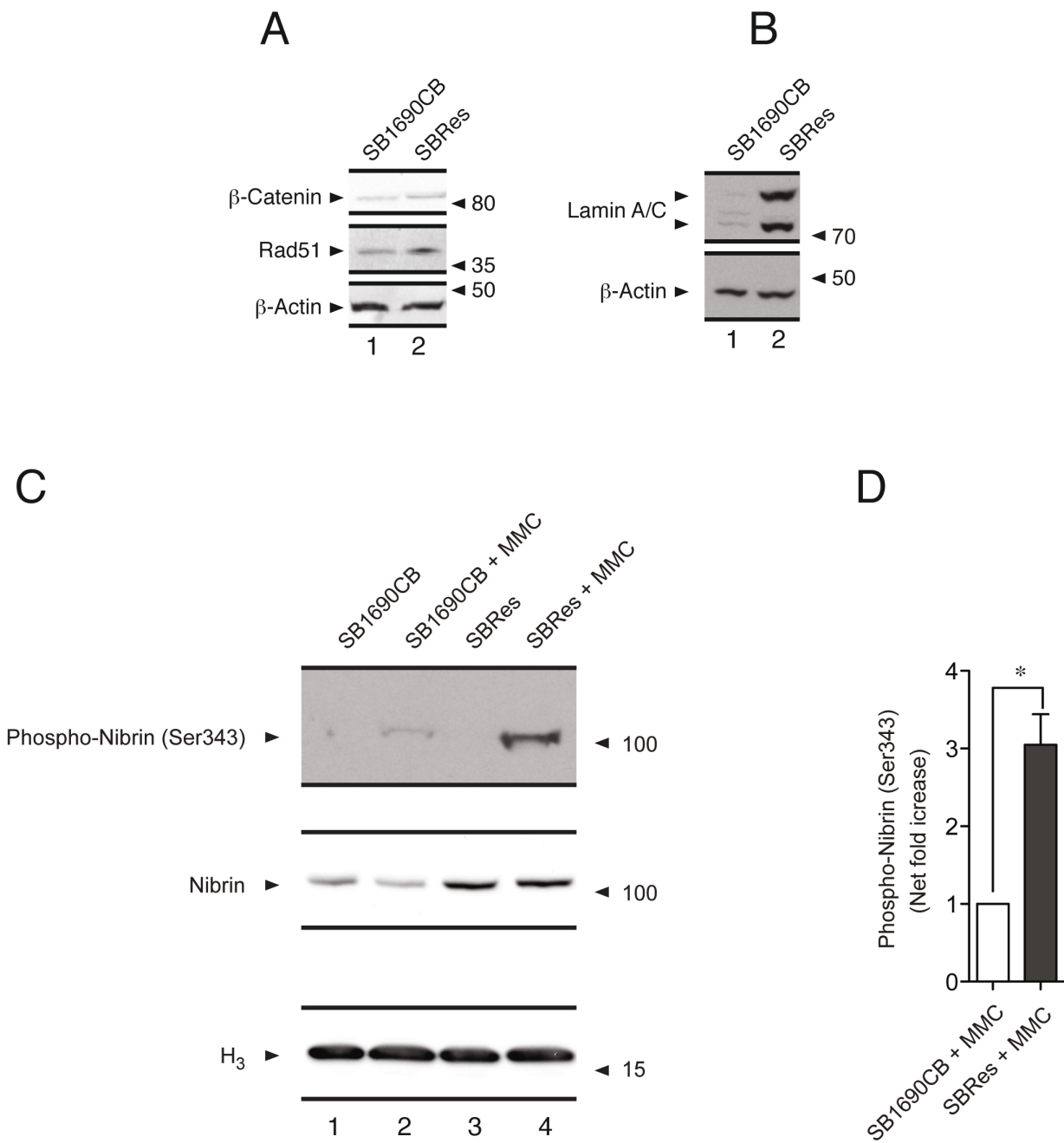

Supplement: Supplementary Figure S1 [file cddis2017264x1.pdf]
