# Supplementary material for: Acquired cross-linker resistance associated with a novel spliced BRCA2 protein variant for molecular phenotyping of BRCA2 disruption
Source: Cell Death Dis. 2017 Jun 15;8(6):e2875–. doi: 10.1038/cddis.2017.264 (PMC5520920; doi:10.1038/cddis.2017.264)

Suppl. Figure 2

A

SB1690CB vs SBRes Network Cluster Hierarchy

CDK2	HDAC1	BRCA1	CTNNB1	BRCA2	HSP90AA1	AR	UIMC1	CUL4A	VCP	IKBKG	SMARCC1	NPM1	YWHAZ	UBE2I	NOTCH1	SSRP1	CDC42	BMI1	RHOA
CDKN1A	HDAC2	H2AFX	BTRC	RAD51	HSP90AB1	NCOR1	BRCC3	CUL4B	NSFL1C	RIPK1	SMARCA2	CDKN2A	TNFAIP3	SMAD4	RBPJ	SUPT16H	IQGAP1	PHC1	ARHGDIA
PCNA	SIN3A	MDC1	PSEN1	BRCA1	NR3C1	HSP90AA1	BABAM1	DDB2	ATXN3	FADD	SMARCB1	NCL	TNIP1	PIAS1	SNW1	H3F3A	CALM1	USP7	CDC42
RPA1	RBBP4	EP300	SMAD4	PALB2	HDAC6	HSP90AB1	BRCA1	COPS3	UBL4A	YWHAZ	SMARCE1	CDK6	YWHAG	RANBP2	JAG1	MCM3	PAK4	SFMBT1	ARHGEF12
RB1	KDM1A	RPA1	CDC34	H2AFX	HSPA1A	HSPA4	MDC1	C1QBP	RAD23B	TNFAIP3	ACTL6A	LMNA	IKBKG	PIAS2	RUNX3	MCM4	ARHGDIA	RYBP	PLD1
EP300	RB1	BRIP1	YWHAZ	FANCD2	FKBP4	HSPA1A	H2AFX	TUBB	UBQLN1	CYLD	BMI1	C1QBP	MARK3	SKIL	TNPO1	NEK9	CDC42EP1	HIST2H2AA3	BID
SKP2	EP300	MRE11A	EZH2	MDC1	HSPA4	HDAC6	USP7	HSPA1A	HSPA4	TNFRSF10B	KAT5	YWHAZ	TRAF3	DNMT3A	DHX8	MCM6	PAK3	CALM1	ARHGEF2
CCNB1	EZH2	PRKDC	AR	RPA1	UBL4A	KAT5	FANCG	HSPA5	GTF3C4	TRAF3	SMARCD2	UBL4A	VIM	GLUL	DHX15	MCM5	LRP6	PRKRA	TEC
CCNA1	WHSC1	TP53BP1	MUC1	FANCG	SUGT1	CTNNB1	EP300	DCAF4	YWHAZ	RIPK3	BAZ1B	AURKA	TRAF1	BMI1	GFI1B	FYTTD1	MYLK	SOCS2	ITPR1
HDAC1	YY1	MLH1	YAP1	BLM	PPP5C	NR3C1	PRKDC	DCAF5	GTF3C5	YWHAG	PBRM1	PPP1CC	YAP1	UBE2K	IPO5	POLR1A	PLD1	YARS2	BIN1

FA vs Normal Network Cluster Hierarchy

UBC	CDK2	CDK4	SUMO2	BARD1	XRCC6	RBBP4	TCEB1	EZH2	IRAK1	CRK	RCOR1	CASP3	RAF1	MAPK14	BUB1B	NBN	MLH1	BCL2L1	MAP1LC3B
SUMO2	UBC	SUMO2	ELAVL1	BRCA1	XRCC5	HDAC1	VHL	EED	TRAF6	CBL	KDM1A	XIAP	RALGDS	MAPK8IP2	CDC20	MRE11A	PMS2	BAD	GABARAPL2
ELAVL1	SUMO2	UBC	UBC	RBBP8	SUMO2	BCL6	CUL5	SUZ12	IRAK3	EGFR	HDAC2	DTX3	HRAS	ATF2	BUB3	MDC1	EXO1	BAX	SQSTM1
PCNA	ELAVL1	ELAVL1	SKP2	UBE2E1	PRKDC	RCOR1	CUL2	PHF1	MYD88	BCAR1	HDAC1	TBK1	HSP90AA1	MAPKAPK2	CDC27	RAD50	BLM	BAK1	ATG4B
USP7	PCNA	RB1	CUL1	UBE2D1	PARP1	HDAC2	TCEB2	SRSF3	IRAK4	GRB2	KIAA0182	UBE2E1	YWHAZ	MAP2K6	MAD2L1	H2AFX	BRIP1	BECN1	ATG7
EIF4A3	CDKN1A	CEBPE	EIF4A3	UBE2D3	ERG	YY1	LAMP2	JARID2	TOLLIP	KIT	SUMO2	CRYAB	YWHAB	MAP2K3	CDC23	TOPBP1	PMS1	BCL2L11	ATG3
PSMA2	SKP2	RBBP4	PSMA2	NBN	ILF2	SUMO2	HNRNPA2B1	SNRPD1	PELI1	PIK3CB	KDM5B	BIRC2	YWHAQ	MKNK1	CASC5	ATM	FAN1	BID	NIPSNAP1
SRPK1	CDK4	CDK2	EIF6	MLH1	WRN	KDM5B	HIF1A	ELAVL1	IKBKG	SOS2	GFI1	CASP9	MAP2K1	DUSP10	ANAPC4	MLH1	MSH6	BIK	NBR1
TUBB	CDKN1B	CCND1	ILF2	HNRNPD	MDM2	EZH2	ASB9	SRSF7	IL1R1	MUC1	ZNF217	CASP8	KRAS	DUSP16	PLK1	TERF2	MSH2	VDAC1	ULK1
ILF2	EIF4A3	USP7	SHFM1	SRSF1	H2AFX	PHB	HNRNPD	MTF2	TAB2	PIK3R1	CTBP2	APAF1	RPS6KA2	LRP2	SNCG	EP300	AIFM1	HRK	KBTBD7

The most central 10 genes of each network cluster ordered from left to right

Overlapping gene

B

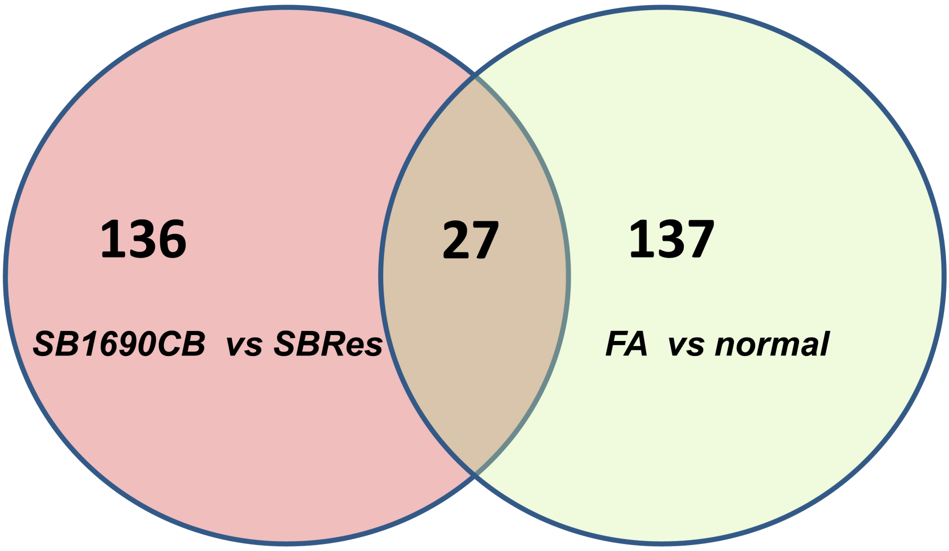

Supplement: Supplementary Figure S2 [file cddis2017264x2.pdf]
