# Supplementary material for: Acquired cross-linker resistance associated with a novel spliced BRCA2 protein variant for molecular phenotyping of BRCA2 disruption
Source: Cell Death Dis. 2017 Jun 15;8(6):e2875–. doi: 10.1038/cddis.2017.264 (PMC5520920; doi:10.1038/cddis.2017.264)
Supplement: Supplementary Information [file cddis2017264x3.docx]

**Supplementary methods**

***Acquired cross linker resistance associated with a novel spliced BRCA2 protein variant for molecular phenotyping of BRCA2 disruption***

S Meyer et al

**Mass spectrometry**

Mass spectrometric analysis was carried as described previously ^[1-3](#_ENREF_1)^. In brief, nuclear protein extracts from two biological replicates were quantified using the modified Bradford protein assay (Bio-Rad). Protein (100 μg) in 30 μl of 1 M TEAB buffer was reduced by addition of 3 μl 50 mM tris-(2-carboxyethyl) phosphine (TCEP) and incubation at 60 °C for one hour. Reduced cysteine residues were blocked by addition of 1.6 μl of 200 mM methylmethanethiosulphate (MMTS) in isopropanol and incubation at room temperature for a further 10 minutes. Proteins were then digested by addition of 5 μl of trypsin at 2 μg/μl in 1 M TEAB and incubated at 37 °C overnight. To label the peptides with iTRAQ reagent (Applied Biosystems), one unit of label (defined as the amount required to label 100 μg of protein) was thawed and reconstituted in 70 μl of isopropanol. The reagent solution was added to the digest and incubated at room temperature for two hours. Labelling reactions were then pooled prior to further processing. For enrichment of phosphopeptides iTRAQ labelled peptide mixtures were re-suspended in 150 µl of lactate loading buffer (240 mg/mL lactate in 80 % (v/v) acetonitrile, 1 % (v/v) TFA). TiO_2_ columns (TopTip, Glygen Corp) were equilibrated with 150 µl of lactate loading buffer then the samples loaded. The tip was washed twice with 60 µl of lactate loading buffer and four times with 60 µl of wash buffer (80 % (v/v) Acetonitrile, 5 % (v/v) TFA, 15 % (v/v) water). Bound peptides were then recovered by elution in 60 µl of elution buffer (ammonium water (20 μl NH_3_ in 980 µl H_2_O), pH 10.5). Samples were then concentrated to a few microliters in a SpeedVac concentrator. Prior to reverse phase LC-MS/MS peptides were fractionated off line using a reverse phase chromatography column (Fortis technologies, C18 3μm 100/4.6 Reverse phase column) at a high pH using an LC Packings Ultimate LC system. The gradient was run at 700 µl/min using initially 99.5% high pH buffer A (0.1% Ammonium hydroxide) 0.5% high pH buffer B (0.1% ammonium hydroxide 99.9% acetonitrile). Over a 30 minute time period high pH buffer B was increased to 50%, followed by 4 minutes to increase high pH buffer B to 75% and then reduced back down to 0.5%. Fifteen second fractions were collected for the duration of the gradient, and then concentrated. Peptides were identified by RP-LC-MS/MS on a QStar^®^ XL mass spectrometer (Applied Biosystems). Dried peptide fractions were re-suspended in 2 % (v/v) acetonitrile/0.1 % (v/v) formic acid. For each analysis, 50% of the peptide sample was loaded onto a 15 cm reverse phase C18 column (75 mm inner diameter) packed with C_18_ PepMap100 (3 µm, 100 Å) using an LC Packings UltiMate^TM^ pump and separated as described previously [^3^](#_ENREF_3). Briefly, peptides were separated over a 120 min solvent gradient from 5.9% (v/v) acetonitrile/0.1% (v/v) formic acid to 41% (v/v) acetonitrile/0.1% (v/v) formic acid on-line to a QStar^®^ XL mass spectrometer (Applied Biosystems). Data was acquired using an information dependant acquisition (IDA) protocol where, for each cycle, the two most abundant multiply charged peptides (2^+^ to 4^+^) above a 20 count threshold in the MS scan with m/z between 400 and 2000 were selected for MS/MS. Each peptide was dynamically excluded (± 50 mmu) for 1 minute. MS data was processed by a 'thorough' search against the Ensembl database release 58 using Protein Pilot 3 software (Paragon version 3.0.0.0,113442) with default settings (Applied Biosystems). ProteinPilot quantification was used for the proteome experiments. For the phosphorylation experiments, identifications with a phosphorylation were selected from the search results using software developed in-house. The ProteinPilot calculation used to derive a weighted average ratio for proteins (ProteinPilot Online Help pp 52-78) was used to derive an average where multiple spectra were found for a given distinct phospho-entity (that is, a distinct sequence and set of modified amino acids). Proteins or peptides were called as changing or differentially expressed when they were detected with a ratio outside the range in which 95% of protein ratios for the internal replicate are found and a p-value of 0.05 or less in at least one experiment

For selective reaction monitoring [^4^](#_ENREF_4) for detection of specific BRCA2 peptides from 25x10^6^ cells were pelleted at 300xg for 4 min (4ºC) and washed 2 times in cold PBS. After removal of cytoplasmic, organelle and membrane protein fractions, remnant protein from pellets was extracted with 10 volumes of a high salt lysis buffer [420 mM NaCl, 20 mM Bicine (Sigma B3876), 2mM MgCl_2_, 1nM ZnCl_2_, 1nM CaCl_2_, 0.6% CHAPS (Sigma C9426), 1mM Na_3_VO_4_, 1mM PMSF, 1X Protease Inhibitor Cocktail (Sigma P8340), 1X Phosphatase Inhibitor Cocktail 2 (Sigma P5726), 1X Phosphatase Inhibitor Cocktail 3 (Sigma P0044) and 250U/µL Pierce Universal Nuclease (Thermo Scientific 88700)] to extract nuclear and chromatin associated proteins. Extracts were subjected to electrophoresis using NuPAGE® Novex® 4-12% Bis-Tris Protein Gels (Life Technology). Proteins were visualised with Instant Blue (Expendon) and proteins larger than 200 kDa were extracted from gel fragments. After tryptic digestion peptides were reconstituted in loading buffer (2% (v/v) acetonitrile (ACN), 0.1 % (v/v) formic acid (FoA), 20mM citric acid) and separated online to the mass spectrometer using a nanoAQUITY UPLC (Waters, UK). A third of the peptide was loaded onto a trap column (Symmetry C18. Waters, UK) with 3% (v/v) ACN, 0.1 % (v/v) FoA at a flow rate of 7 µl/minute for 5 minutes prior to separation by a reverse phase analytical column, BEH130C18 column of 25 cm length and 1.7 µm particle size (Waters, UK) with elution by a 40 minute gradient running from 3 (v/v) % ACN to 40 (v/v) % ACN with o.1 (v/v) % FoA throughout at a flow rate of 0.3µl. Mass spectrometry analysis was carried out by a QTRAP 6500 instrument (SCIEX) set up to monitor specific fragments of precursors, which had been identified as splice form specific using a SRM method. The precursor monitored was 674.7 with the additional fragment ions scanned (1059.5, 873.4, 744.4, 1174.5, 832.4, 775.8, 732.3, 862.3, 773.8, 853.8). The precursor ion was isolated with low resolution with all fragments ions scanned at unit resolution. The dwell time was set to 50 ms with collision energy of 34.3 for all precusor ions. All SRM data was analysed using skyline version 3.1.0.7382 [^5^](#_ENREF_5).

The entire dataset is available at [**http://www.scalpl.org/public/meyerbrca2reversion/**](http://www.scalpl.org/public/meyerbrca2reversion/)**.**

1. Pierce A, Unwin RD, Evans CA, Griffiths S, Carney L, Zhang L *et al.* Eight-channel iTRAQ enables comparison of the activity of six leukemogenic tyrosine kinases. *Mol Cell Proteomics* 2008; **7**(5)**:** 853-63.

2. Unwin RD, Smith DL, Blinco D, Wilson CL, Miller CJ, Evans CA *et al.* Quantitative proteomics reveals posttranslational control as a regulatory factor in primary hematopoietic stem cells. *Blood* 2006; **107**(12)**:** 4687-94.

3. Unwin RD, Pierce A, Watson RB, Sternberg DW, Whetton AD. Quantitative proteomic analysis using isobaric protein tags enables rapid comparison of changes in transcript and protein levels in transformed cells. *Mol Cell Proteomics* 2005; **4**(7)**:** 924-35.

4. Unwin RD, Griffiths JR, Whetton AD. A sensitive mass spectrometric method for hypothesis-driven detection of peptide post-translational modifications: multiple reaction monitoring-initiated detection and sequencing (MIDAS). *Nature protocols* 2009; **4**(6)**:** 870-7.

5. MacLean B, Tomazela DM, Shulman N, Chambers M, Finney GL, Frewen B *et al.* Skyline: an open source document editor for creating and analyzing targeted proteomics experiments. *Bioinformatics* 2010; **26**(7)**:** 966-8.
